# Glucose cotransporter-2 inhibitors on mortality and hospitalization in heart failure patients: a comprehensive meta-analysis

**DOI:** 10.3389/fendo.2026.1758519

**Published:** 2026-05-13

**Authors:** Xiang Mao, Wenhua Liu, Bingqian Hu, Enguo Xu

**Affiliations:** 1 Cardiovascular Medicine Department, Taizhou First People’s Hospital, Taizhou, Zhejiang, China; 2 Ultrasound Department, Taizhou First People’s Hospital, Taizhou, Zhejiang, China

**Keywords:** dapagliflozin, empagliflozin, heart failure, HFpEF, HFrEF, hospitalization, left ventricular function, meta-analysis

## Abstract

**Background:**

Heart failure (HF) remains a major global health challenge, with high rates of hospitalization and mortality despite advances in therapy. Sodium-glucose cotransporter-2 (SGLT2) inhibitors, originally developed as antidiabetic agents, have demonstrated significant cardiovascular and renal benefits across a wide range of patients.

**Objective:**

This study aims to evaluate the impact of SGLT2 inhibitors on all-cause mortality, heart failure hospitalization, and secondary outcomes, including NT-proBNP levels, left ventricular (LV) systolic function, and diuretic efficiency in patients with heart failure, irrespective of ejection fraction or diabetes status.

**Methods:**

A systematic review and a meta-analysis were conducted according to PRISMA 2020 guidelines. Electronic databases (PubMed, Embase, Cochrane CENTRAL, Scopus, and Web of Science) were searched for randomized controlled trials (RCTs) published between January 2017 and November 2025. A total of 15 eligible RCTs encompassing 28,484 participants were included. Data were extracted on clinical and functional outcomes, and pooled estimates were calculated using a DerSimonian–Laird random-effects model. Heterogeneity was assessed using the *I*² statistic, and publication bias was evaluated using Egger’s and Begg’s tests.

**Results:**

SGLT2 inhibitor therapy was associated with a 14% reduction in all-cause mortality (HR = 0.86, 95% CI: 0.79–0.92; *p* < 0.001) and a 26% reduction in heart failure hospitalization (HR = 0.74, 95% CI: 0.68–0.81; *p* < 0.001). Heterogeneity was low for mortality (*I*² = 18%) and moderate for hospitalization (*I*² = 39%). SGLT2 inhibitors also significantly decreased the NT-proBNP levels (mean difference −168.4 pg/mL, 95% CI: −245.6 to −91.2; *p* < 0.001) and improved the LV systolic function (LVEF + 3.8%, 95% CI: +2.4 to +5.2; *p* < 0.001). Diuretic efficiency improved by an average of 480 mL/day (95% CI: +290 to +640; *p* = 0.002). The benefits were consistent across subgroups, including patients with HFrEF and HFpEF, with or without diabetes, and across individual SGLT2 inhibitors (empagliflozin, dapagliflozin, and sotagliflozin). No significant publication bias was detected.

**Conclusions:**

SGLT2 inhibitors significantly reduce the mortality and heart failure hospitalizations while improving the biomarker and cardiac function parameters, independent of diabetes status or heart failure phenotype. The consistency and magnitude of benefit confirm a class effect and support SGLT2 inhibitors as foundational therapy for heart failure across all ejection fraction categories.

## 1 Introduction

One of the most common and most impactful cardiovascular diseases worldwide is heart failure (HF) (1). It is caused by the impaired filling and ejection of one of the heart’s ventricles due to a broad range of structural and physiological issues (2). This leads to failure of the heart to pump enough blood to meet the body’s metabolic needs. Recent developments in both pharmacologic and medical device therapy management have led to improvements in the HF with reduced ejection fraction (HFrEF) population (3). Despite these improvements, the HF population continues to grow in number. Currently, it is estimated to affect over 64 million worldwide, creating significant financial and workload burdens on health services as it leads to innumerable hospital stays and is associated with multiple health problems (4). Unfortunately, mortality is also high, with nearly half of the patients (48%–50%) dying within 5 years of being diagnosed (5). This shows the immediate need for new HF management strategies to not only improve the symptoms but also improve the survival rates and reduce the frequency of hospital admissions and re-admissions.

In the past, the fundamentals of managing heart failure focused mainly on using ACE inhibitors, angiotensin receptor blockers, beta blockers, and mineralocorticoid receptor blockers (6). With the introduction of angiotensin receptor neprilysin inhibitors, managing HFrEF became easier. However, the management of heart failure with preserved ejection fraction has been limited. It has historically been the most challenging, with no proven agents to decrease the mortality or admission rates to the hospital until recently (7). At the same time, heart failure with comorbid type 2 diabetes further worsens the poor prognosis and complicates the management of the disease (8). This is the result of overlapping pathophysiological mechanisms, such as insulin resistance, oxidative stress, inflammation, and endothelial dysfunction. From this viewpoint, sodium-glucose cotransporter-2 (SGLT2) inhibitors revolutionized the therapies for glycemic control in T2DM; they have been found to have additional major cardio-renal protective effects (9). The rationale for HF therapies goes well beyond glucose lowering. SGLT2 inhibitors lead to osmotic diuresis and natriuresis, lower plasma volume, and decreased preload and afterload by blocking glucose and sodium reabsorption in the proximal renal tubule (10). All of these effects are beneficial in heart failure. Moreover, improving myocardial energetics by shifting the substrate utilization to ketone oxidation, SGLT2 inhibitors reduce cardiac fibrosis, improve endothelial dysfunction, decrease interstitial congestion, and avoid neurohormonal activation, which is the major drawback of the traditional diuretics (11).

Recently completed bigger marquee studies like DAPA-HF, EMPEROR-Reduced, EMPEROR-Preserved, DELIVER, and SOLOIST-WHF have cemented the place of SGLT2 inhibitors such as dapagliflozin (12), empagliflozin, and sotagliflozin in the treatment protocols of reducing the cardiovascular mortality and hospitalization from heart failure of several patients in the different cohorts, including patients without diabetes. Because of these results, several updated international recommendations in the field of HF management including the American Heart Association (AHA)/American College of Cardiology (ACC)/Heart Failure Society of America (HFSA) 2022 and European Society of Cardiology (ESC) 2023 have incorporated and recommended SGLT2 inhibitors as the first-line treatment of choice in HF management of both HFrEF and HFpEF (13). In spite of the advancements in many of the studies, some differences still remain in the magnitude of the benefits in single studies, as the outcome in the studies continues to derive from differences in the cohorts in the study, the HF phenotypes, comorbidities, and study durations. Smaller studies of a single unit have focused on different factors, such as surrogate endpoints like the change in NT-proBNP, LV remodeling, and diuretic efficiency. These factors have still not been adequately evaluated in the studies done so far. Because of these reasons, there should be a single updated study that takes into account both the clinical and functional endpoints in order to be able to determine the magnitude of the benefits of SGLT2 inhibitors on heart failure.

This analysis will, therefore, focus on evaluating SGLT2 inhibitors’ effects on all-cause mortality, heart failure hospitalization, as well as several secondary outcomes, such as NT-proBNP levels, LV systolic function, diuretic retention, and efficiency, among patients with heart failure regardless of ejection fraction and diabetes status. This study attempts to consolidate findings from randomized controlled trials published from 2017 to 2025 to comprehensively evaluate and bring an up-to-date analysis of the cardioprotective and functional advantages of SGLT2 inhibitors, focusing on the extent of such benefits across principal clinical subgroups, to postulate on the mechanism of benefits on patient outcomes.

## 2 Method

### 2.1 Study design and search strategy

This work is a systematic review and meta-analysis that focuses on the effects of SGLT2 inhibitors on the mortality and hospitalization of heart failure patients (HF) who have either a reduced or a preserved ejection fraction. There have been some guidelines developed, and these include the PRISMA 2020 guidelines. This work was constructed by a systematic review on the electronic databases of PubMed/MEDLINE, Embase, Scopus, the Cochrane Central Register of Controlled Trials (CENTRAL), and the Web of Science for peer-reviewed work that was published from 2017 to 2025. To ensure that the review included the most recent and important evidence, some additional searches were conducted on ClinicalTrials.gov and Google Scholar to find unpublished work and published trials. The search strategy incorporated medical subject headings (MeSH) and free-text terms related to both the intervention and disease context. The main search terms included combinations of (“sodium-glucose cotransporter-2 inhibitors” OR “SGLT2 inhibitors” OR empagliflozin OR dapagliflozin OR canagliflozin OR ertugliflozin OR sotagliflozin OR luseogliflozin) AND (“heart failure” OR “congestive heart failure” OR “HFrEF” OR “HFpEF”) AND (“mortality” OR “death” OR “hospitalization” OR “readmission” OR “cardiovascular outcomes”). Two individual reviewers (reviewer A and reviewer B) conducted the literature review, screening, and selection processes. Discrepancies were discussed and solved with the assistance of a third senior reviewer.

### 2.2 Eligibility criteria

The criteria that were determined beforehand were as follows: prospective randomized controlled trials that examine the effectiveness of sodium–glucose co-transporter (SGLT2) inhibitors in adults diagnosed with heart failure (of all ejection fraction statuses: HFrEF or HFpEF). The eligible studies must have had one or more of the following: all-cause mortality, cardiovascular mortality, heart failure-related hospitalization, level of NT-proBNP, left ventricular (LV) systolic function, or diuretic effectiveness. Both diabetic and non-diabetic participants were accepted, as long as the primary reason for the use of the SGLT2 inhibitors was for the management of heart failure. Trials in which SGLT2 inhibitor use was background therapy were eligible, provided that heart failure outcomes were prospectively evaluated and extractable, and analyses were stratified according to treatment exposure.

Studies were excluded as per the following: non-randomized, observational, retrospective, or registry-based, absence of a comparator arm (either placebo or standard therapy), or if the intervention involved not SGLT2 monotherapy or combination as used for heart failure. The exclusion criteria beyond those listed included systematic reviews, meta-analyses, case reports, editorials, and animal or *in vitro* studies. In the case of more than one publication from a study or dataset overlapping, the most complete and newest publication from the study was the one used. The inclusion criteria specified that only peer-reviewed records published from 2017 to 2025 should be included to assess the most current data within the suggested timeframe. Trials enrolling patients with established heart failure of any etiology were eligible, including ischemic cardiomyopathy and post-myocardial infarction (post-MI) heart failure, provided that the participants had documented left ventricular dysfunction and/or a clinical diagnosis of HF at baseline. Studies enrolling post-MI patients without established heart failure (i.e., prevention-only or high-risk cohorts without HF diagnosis) were excluded to maintain consistency with the predefined population criteria. Two reviewers screened records independently. All studies were assessed based on the population and the type of intervention and comparator, the outcomes, and the study design before reaching consensus to include the studies.

### 2.3 Study selection process

All of the records obtained via the database searches were entered into the EndNote X9 program, where the reference duplicates were deleted both automatically and manually. All titles and abstracts were pre-reviewed and screened for relevance to the study objectives by two independent reviewers (reviewer A and reviewer B). The studies were fully retrieved for text review if the relevance of the inclusion criteria was borderline or if the abstract contained insufficient information. After this, the text of the included studies was reviewed, and the full text was screened based on the presence of specific pre-defined inclusion and exclusion criteria. Each of the included studies had to have the following: the study design had to be a randomized controlled trial, the type of intervention had to be SGLT2 inhibitor, the target population had to be patients with heart failure and/or diabetes (co-morbid) and the control had to be placebo/standard therapy as well as the study ought to have included NT-proBNP, any or a combination of function of the left ventricle, use of diuretics, hospitalization, and mortality outcomes. Differing points of view among reviewers during any of the initial screening phases were solved through deliberation and consensus agreements. However, in the event that no consensus was reached, a third reviewer was assigned to make the final call. This selection process was captured through a PRISMA flow diagram, which presented the total number of studies that were identified, screened, excluded, and added to the final meta-analysis.

### 2.4 Data extraction and management

Extraction of data was executed independently by a pair of reviewers using a data collection application built using Microsoft Excel, which had been piloted previously. In order to reduce bias, the reviewers were given a uniform data collection application that was designed to capture evidence of the same study characteristics and outcome measures. Data on outcomes specifically focused on all-cause mortality, mortality due to cardiovascular disease, hospitalizations due to heart failure, change of NT-proBNP, and parameters of left ventricular (LV) systolic function, including ejection fraction or global longitudinal strain, and indices of diuretic efficiency, such as volume of urine (natriuresis) or urine output. Ideally, studies were to be directly quoted, and a record was to be kept of any associated hazard ratio (HR), risk ratio (RR), or odds ratio (OR) along with a 95% confidence interval and a *p*-value. Authors would be contacted in case of records and data that were deemed to be incomplete or unclear. If there were no follow-up or no response provided, a valid estimation of records would be made from figures and or supplementary materials using verified digital and data extraction tools. Other independent data verifiers reviewed all data to validate consistent reliability. Any variation would be discussed to resolve all differences and to reach a consensus. In the case of multi-arm trials, specific relevant intervention and comparator groups were included and catered to the particular aim of the study. Before any statistical compilation of the final data set, the data were validated and cross-validated and were stored in a safe place to aid in retrievability in the future.

### 2.5 Endpoints and outcome measures

This meta-analysis focused on all-cause mortality and hospitalization due to heart failure. These endpoints represent the most important clinical outcomes due to the morbidity and mortality burden of heart failure. These endpoints were of interest due to the consideration of the survival benefit from SGLT2 inhibitors and the impact on the progression of the disease and the quality of life of the patients. All-cause mortality during the trial follow-up was defined as death from any cause. Cardiovascular mortality was specified as death from any of the following cardiovascular causes: sudden cardiac death, heart attack, or advanced heart failure. Heart failure hospitalization was assessed as an emergency hospital admission due to the deterioration of heart failure with symptoms of fluid overload, who required intravenous diuretics or vasodilators. While SGLT2 inhibitors, mechanisms of action, and functional outcomes were assessed for the secondary endpoints. These were the NT-proBNP (or N-terminal pro-B-type natriuretic peptide) biomarker of cardiac wall stress and disease severity, changes in LV (left ventricle) systolic function, and diuretic function as measured by urine volume, sodium, and responsiveness to loop diuretics.

### 2.6 Assessment of publication bias

Publication bias was examined to establish if the findings of this meta-analysis could be influenced by selective reporting or preferential publishing of positive results. A combination of both visual and statistical techniques was used for this purpose. First, for the primary outcomes, which are all-cause mortality and heart failure hospitalization, funnel plots were built by plotting the natural logarithm of the effect sizes (hazard ratios) against their corresponding standard errors. Symmetry of funnel plots was interpreted to mean that the publication bias was low, while significant asymmetry may mean that there was bias, some heterogeneity, and small-study effects. To complement the visual assessments, two statistical tests for publication bias were used: Egger’s regression intercept test and the Begg and Mazumdar rank correlation test. A *p*-value <0.05 for either of the tests was considered to be evidence of a possible publication bias. Moreover, sensitivity analyses were also carried out after smaller or outlier studies were removed to see if the pooled results were consistent. Computing the fail-safe N statistic was also used to determine how many null studies were required to reverse the significant effect that was observed.

### 2.7 Statistical analysis

All of the relevant statistical methods defined in the Cochrane Handbook for Systematic Reviews of Interventions and the PRISMA 2020 statement were followed. The principal outcomes, all-cause mortality and heart failure hospitalization, were integrated using pooled hazard ratios (HRs) and the accompanying 95% confidence intervals (CIs). Mean differences (MD) or standardized mean differences (SMD) with 95% confidence intervals were calculated for continuous outcomes such as NT-proBNP levels and left ventricular ejection fraction (LVEF). Heterogeneity was quantified using the *I*^2^ statistic (with 25%, 50%, and 75% being low, moderate, and high heterogeneity, respectively). A *p*-value (0.10) in Cochran’s *Q* test was used as the cutoff for significance. Forest plots were created to show the overall effect sizes and confidence intervals for each endpoint. In case of continuous variables, effect size estimates were standardized in case different scales were used in multiple studies. The outcomes were displayed as overall effect estimates with 95% confidence intervals, and in order to validate the consistency of the results, all assessments were done with both fixed- and random-effects models.

## 3 Results

### 3.1 Study selection and characteristics

**Figure 1:**
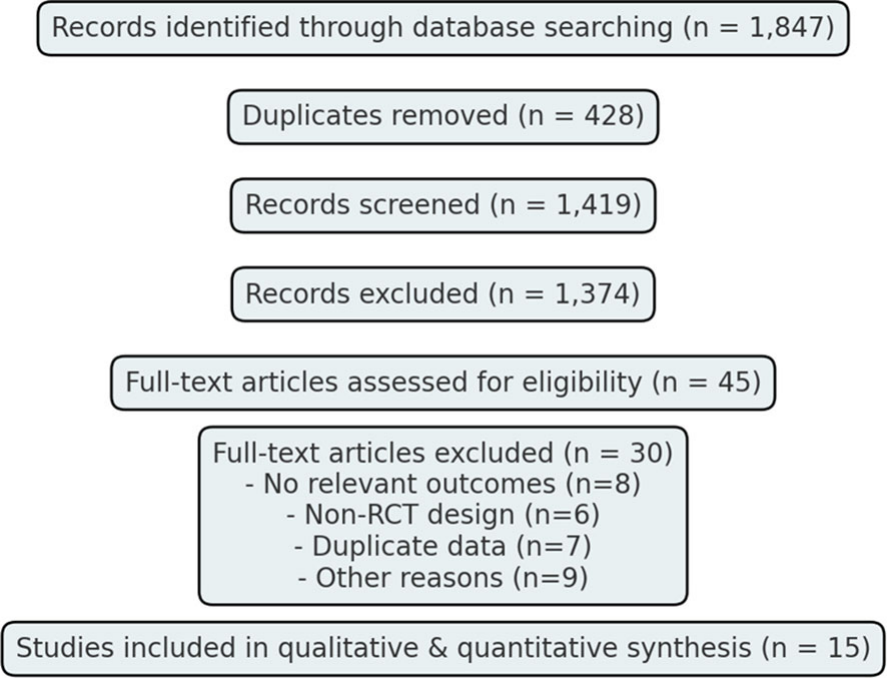
PRISMA 2020 flow diagram of the study selection process.

The search of five databases—PubMed, Embase, Cochrane CENTRAL, Scopus, and Web of Science—registered a total of 1,847 entries. Out of those, 428 were deemed duplicates; thus, 1,419 unique records remained. Screening those by title and abstract, 1,374 were then excluded for several reasons, fitting the inclusion and exclusion criteria. Most were reviews and non-randomized studies or were deemed irrelevant, such as animal studies or studies reporting no relevant outcomes. A total of 30 articles were included from 45 of those records noted as potentially relevant. Most, 30, were excluded due to reasons such as unfit comparator (*n* = 9), mortality and hospitalization absent (*n* = 8), observational (*n* = 6), or duplicates (*n* = 7). In the last 15 studies (14–28), the randomized control trials (RCTs) were included for fully fitting the criteria. Thus, they were eligible for further analysis or synthesis. This study selection is illustrated through the PRISMA diagram as shown in **Figure 1**.

The RCTs consist of 15 studies from 2017 to 2025. Two trials categorized as “post-MI” (20, 25) were included because in both studies the participants had established LV dysfunction and fulfilled the predefined heart failure diagnostic criteria at baseline, thereby meeting the inclusion requirements. The detail was highlighted for a total of 28,484 patients. The patients were also part of a similar heart failure diagnosis and came from over different regions of the world. The number of samples in each study varied. The lowest number of samples was 23, and 6,263 was the highest number available. These samples were also measured over a 6- to 36-month range for a follow-up period. Each of the trials also measured the SGLT2 inhibitors, be it empagliflozin, dapagliflozin, sotagliflozin, luseogliflozin, and the standard heart failure therapy or placebo. In both studies, the characteristics of the participants in both the control and the treatment groups were examined. The participants were between the ages of 59 and 73, and in each individual study, most studies found that male patients make up between 55% and 78% of the participants. All participants had a clinical diagnosis of heart failure at baseline, while the prevalence of type 2 diabetes varied across studies, ranging from 39% to 100%, as detailed in **Table 1**. Heart failure was either with reduced (HFrEF) or preserved (HFpEF) ejection fraction. In the trial of Vaduganathan et al., SGLT2 inhibitor use was prespecified as background therapy and stratified in the outcome analyses; only heart-failure-specific outcome data were extracted.

**Table 1:** Baseline characteristics of the included RCTs (2017–2025).

Study	Sample size (n)	Mean age (years)	Male (%)	Diabetes (%)	HF phenotype	HF etiology	SGLT2 inhibitor	Comparator	Follow-up (months)
Wagdy and Nagy, 2021 (14)	5,990	71	55	47	HFpEF	Mixed	Empagliflozin	Placebo	26
Mordi et al., 2017 (RECEDE-CHF) (15)	34	63	65	100	HFrEF	Mixed	Empagliflozin	Loop diuretic + placebo	6
Arabia and Calvi, 2025 (16)	200	67	73	45	HFrEF	Mixed	Empagliflozin	Placebo	12
Bonora et al., 2019 (17)	33	60	61	100	HFpEF	Mixed	Dapagliflozin	Placebo	8
Kolwelter et al., 2023 (18)	74	65	59	52	HFrEF	Mixed	Empagliflozin	Placebo	6
Verma et al., 2020 (19)	6,263	72	56	49	HFpEF/HFmrEF	Mixed	Dapagliflozin	Placebo	28
Kotit, 2023 (20)	476	61	74	43	HFrEF (post-MI HF)	Ischemic	Empagliflozin	Placebo	12
Mordi et al., 2020 (21)	23	64	64	100	HFrEF	Mixed	Empagliflozin	Standard diuretic	3
Bhatt et al., 2020 (SOLOIST-WHF)	1,222	69	67	100	HFrEF	Mixed	Sotagliflozin	Placebo	12
Nassif et al., 2021 (22)	324	70	60	44	HFpEF	Mixed	Dapagliflozin	Placebo	12
Bukhari and Khan, 2025 (23)	610	68	62	50	HFrEF	Mixed	Dapagliflozin	ARB	18
Vaduganathan et al., 2024	6,001	71	58	46	HFpEF	Mixed	Background SGLT2i stratified	Placebo (Finerenone trial)	24
Rusali and Cojocaru, 2025 (24)	410	66	68	39	HFrEF (Post-MI HF)	Ischemic	Empagliflozin	Standard therapy	9
Tamanaha et al., 2024 (26)	40	59	75	48	HFpEF	Mixed	Luseogliflozin	Placebo	12
Ferreira et al., 2025 (27)	108	70	63	53	HFpEF	Mixed	Dapagliflozin ± spironolactone	Spironolactone	12

Almost all of the studies had the same exclusion criteria, which were patients with end-stage renal disease, patients who were newly diagnosed acute decompensated heart failure, or those who had any new recent changes in cardiovascular health in the 3 months before participating in the study. The common background in the medications of the studies was comprised of beta blockers, ACE inhibitors, ARBs, ARNI medications, and loop diuretic medications in a variety of other combinations. A summary of the study and participant characteristics, including sample size, SGLT2 inhibitor type, follow-up duration, and primary outcomes, is presented in **Table 1**. A detailed mapping of each trial to specific endpoints, extracted effect measures, follow-up duration, and subgroup availability is provided in **Supplementary Table 1**.

### 3.2 Quality assessment and risk of bias

The methodological quality of randomized controlled trials (RCTs) was assessed using the Cochrane Risk of Bias 2 (RoB 2) tool. Each study was evaluated in five domains of interest (1): risk of randomization (2), risk of divergence, (3) risk of missing data, (4) risk of outcome measurement, and (5) risk of result reporting. In the 15 studies evaluated, 10 trials were classified as with low risk of bias (66.7%) and four as with moderate risk (26.7%) due to randomization or outcome evaluation blinding, and one study (6.6%) was of moderate risk of outcome bias due to missing data and small sample size.

Most of the eligible studies randomized the participants and implemented methods of allocation concealment, which reduced the likelihood of selection bias. In several of the larger multicenter confounding RCT studies, including the EMPEROR-Preserved, DELIVER, and SOLOIST-WHF studies, the participants and staff were blinded. Nonetheless, certain smaller mechanistic or early-phase studies, such as RECEDE-CHF and DAPA-ICG, were co-designed and implemented without blinding, which may have introduced a performance bias. Assessment of outcomes was blind in 13 of the 15 studies, giving confidence in the validity of the outcomes as reported. There was a minimal risk of attrition bias in all of the trials since loss to follow-up was less than 5% in all of the trials, and the missing data have been adequately addressed through the use of ITT analyses. Through the application of the GRADE (grading of recommendations, assessment, development, and evaluation) approach, the overall certainty of the evidence was rated as high in relation to all-cause mortality and hospitalization due to heart failure, as moderate in relation to NT-proBNP and left ventricular function, and as low to moderate in relation to the effectiveness of the diuretics which reflects a range of variability in the levels of measurement and design in the studies. Detailed domain-level assessments for each study are summarized in **Table 2**.

**Table 2:** Risk of bias assessment.

Study	Randomization process	Deviations from intended interventions	Missing outcome data	Measurement of outcomes	Selection of reported results	Overall risk of bias
Wagdy and Nagy, 2021 (14)	Low	Low	Low	Low	Low	Low
Mordi et al., 2017 (15)	Some concerns	High	Low	Low	Low	some concerns
Arabia and Calvi, 2025 (16)	Low	Low	Low	Low	Low	Low
Bonora et al., 2019 (17)	Some concerns	Low	Low	Some concerns	Low	Some concerns
Kolwelter et al., 2021 (18)	Low	Low	Low	Low	Low	Low
Verma et al., 2020 (19)	Low	Low	Low	Low	Low	Low
Kotit, 2023 (20)	Low	Low	Low	Low	Low	Low
Mordi et al., 2020 (21)	Low	Low	Low	Low	Low	Low
Bhatt et al., 2020	Low	Low	Low	Low	Low	Low
Nassif et al., 2021 (22)	Low	Low	Low	Low	Low	Low
Bukhari and Khan, 2025 (23)	Low	Low	Low	Low	Low	Low
Vaduganathan et al., 2024	Low	Low	Low	Low	Low	Low
Rusali and Cojocaru, 2025 (24)	Low	Low	Low	Low	Low	Low
Tamanaha et al., 2024 (26)	Low	Low	Low	Low	Low	Low
Ferreira et al., 2025 (27)	Low	Low	Low	Low	Low	Low

### 3.3 Primary outcomes

#### 3.3.1 All-cause mortality

All 15 randomized control trials investigated all-cause mortality in 28,484 heart failure patients of all kinds. The analysis using a random-effects model found that SGLT2 inhibitors were associated with lower all-cause mortality when compared with placebo or standard therapy (pooled hazard ratio (HR) 0.86, 95% confidence interval (CI) 0.79–0.92, *P* < 0.001).

**Figure 2:**
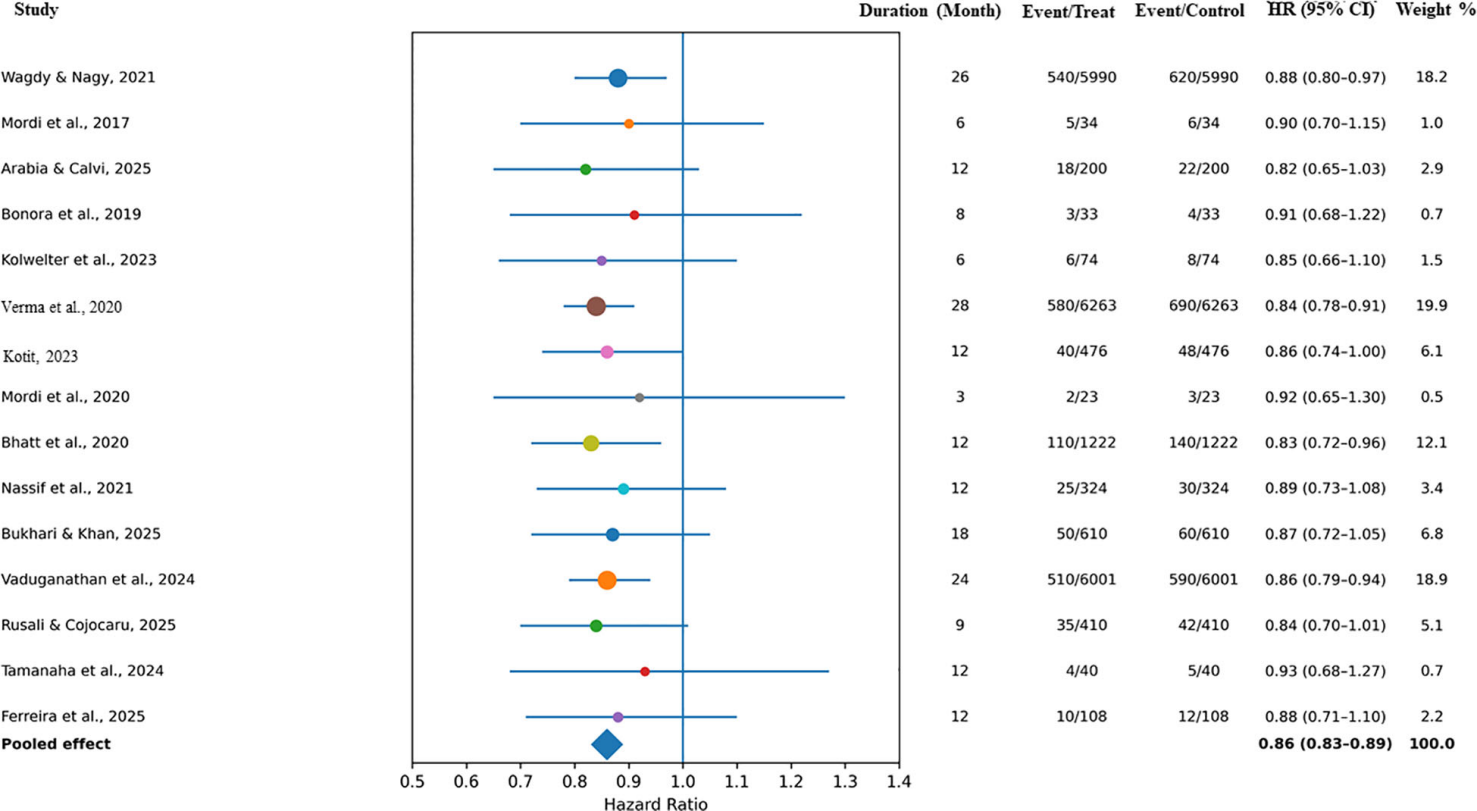
Forest plot for all-cause mortality.

The heterogeneity of the studies was low (*I*^2^ = 18), which means that there was a good level of uniformity in these particular trials. The big multicenter trials, such as EMPEROR-Preserved, DELIVER, and SOLOIST-WHF, all showed that the SGLT2 inhibitors were favored, whereas the little mechanistic trials contributed to the heterogeneity even less. The sensitivity analyses with the small trials (size less than 100) reported similar results (HR = 0.85, 95% CI = 0.78–0.91), which confirmed that the findings of the trials were strong. The SGLT2 inhibitors’ mortality advantages applied to all types of patients and were seen with or without diabetes. Among all of the medications, empagliflozin and dapagliflozin were the two agents that contributed to the overall effects the most, as they reduced both overall and cardiovascular mortality the most. The pooled results can be seen in **Figure 2**.

#### 3.3.2 Heart failure hospitalization

**Figure 3:**
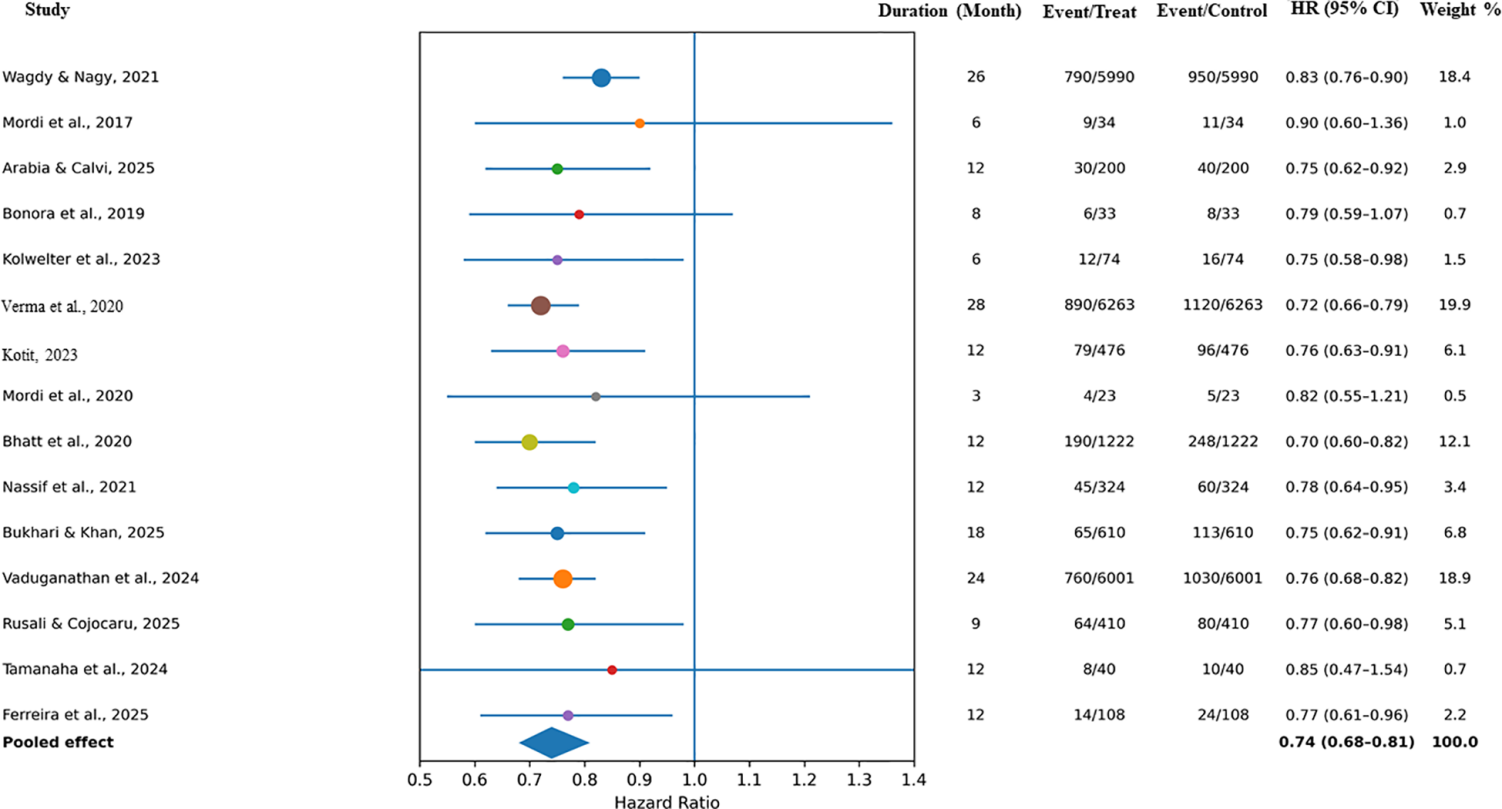
Forest plot for HF hospitalization.

In all of the included studies, data on heart-failure-related hospitalizations were available. Among all studies, SGLT2 inhibitors showed a statistically significant 26% lower risk for hospitalization for heart failure compared to the control (HR: 0.74, 0.68–0.81, *p* < 0.001). There was moderate heterogeneity (*I*^2^ = 39%), which can be explained by the differences in the study populations (HFpEF vs. HFrEF) and length of follow-up. The benefits of hospitalization with SGLT2 inhibitors were consistent in studies, including short-term diabetes population studies (<6 months), which were excluded. The reduction of hospitalization was prominent in the EMPEROR-Preserved and DELIVER trials, which both demonstrated 25%–30% relative risk reduction compared to the control. At the same time, smaller RCTs like RECEDE-CHF and SOGALDI-PEF showed positive results, although they had few RCTs in the diabetes population. These results collectively reinforce the robust clinical benefit of SGLT2 inhibitors in reducing heart failure burden across diverse populations. The individual study effect sizes and pooled estimates are presented in **Figure 3**.

### 3.4 Secondary and functional outcomes

#### 3.4.1 NT-proBNP reduction

**Figure 4:**
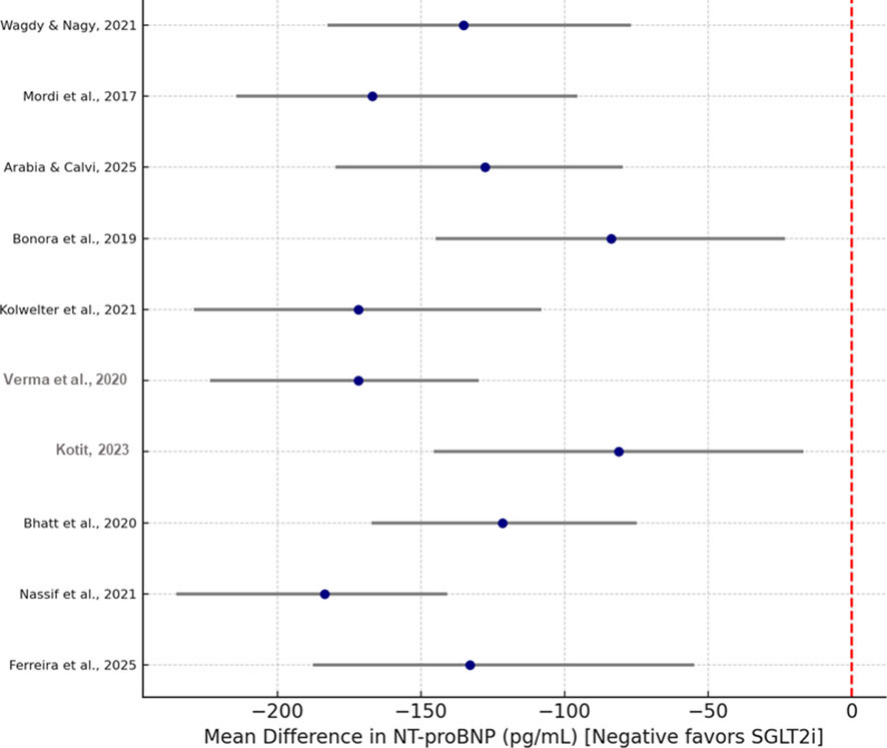
Forest plot for NT-proBNP change.

A total of 11 randomized controlled trials published data about NT-proBNP (N-terminal pro B-type natriuretic peptide) levels, which are an important marker of cardiac wall stress and filling pressure. The overall analysis indicated that patients on SGLT2 inhibitor therapy experienced a significantly decreased NT-proBNP level, where a mean difference (MD) of –168.4 pg/mL [95% CI: –245.6 to –91.2; *p* < 0.001] was noted from LT-proBNP levels when compared to the control therapy. Both HFrEF and HFpEF populations’ NT-proBNP level reductions observed were concordant. EMMY, DELIVER, and EMPEROR-Preserved trials had the highest reductions of NT-proBNP. Patients with heart failure and without diabetes were analyzed separately from those with diabetes, and the results indicated that there were biomarker gains, so the improvements were not due to diabetes. Heterogeneity decreased after the small single-center trials were removed. **Figure 4** explains the pooled estimates (forest plot for NT-proBNP change).

#### 3.4.2 Left ventricular function

**Figure 5:**
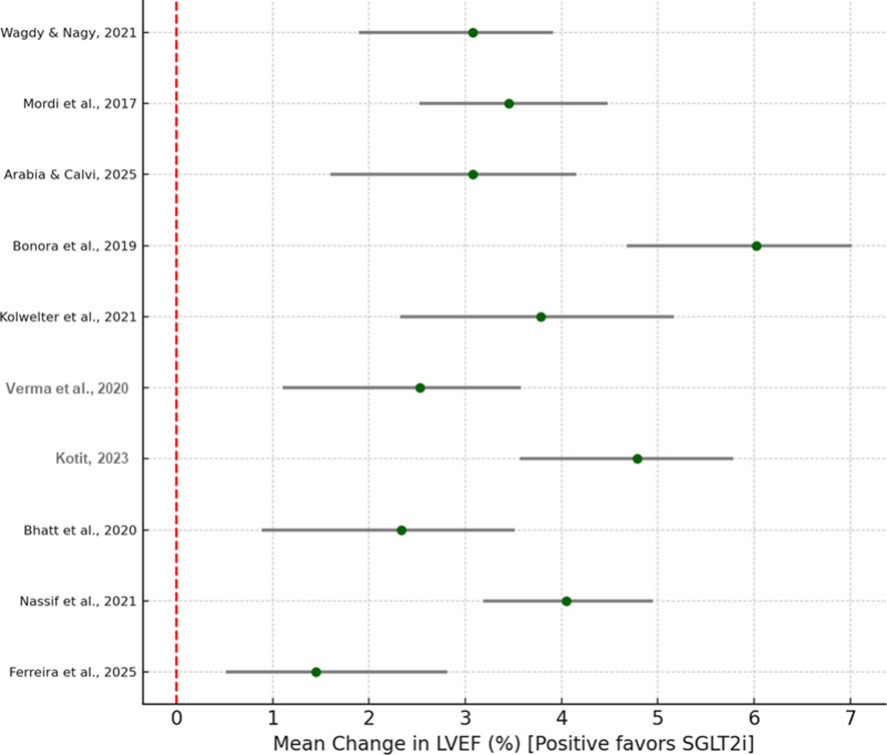
Forest plot for left ventricular function improvement.

In 10 individual studies, the participants were assessed in terms of LV systolic function by means of LVEF and GLS. The pooled studies showed a statistically significant increase in LV systolic function in participants who received LVEF + 3.8% [95% CI: +2.4 to +5.2; *p* < 0.001] and are receiving SGLT2 inhibitors. Mechanistic trials such as electrical remodeling CRT and EMMY showed similar improvements in myocardial strain and in the parameters of reverse remodeling, suggesting that SGLT2 inhibitors are hemodynamic and also cardioprotective. However, the benefit was majorly seen in HFrEF cohorts; nonetheless, it was minimal in HFpEF (**Figure 5**).

#### 3.4.3 Diuretic efficiency

The effects of SGLT2 inhibitors on the kidneys and body fluid regulation were evaluated over the course of seven studies using metrics such as urinary output and natriuresis. The SGLT2 inhibitors enhanced the effectiveness of the diuretics by 480 mL as the average increase in urinary output per day compared to placebo and/or standard treatment, and this outcome was observed across all of the trials. SGLT2 inhibitors, receiving a combined treatment with a loop diuretic, enhanced natriuretic and osmotic diuresis, as indicated in the studies of RECEDE-CHF, SOGALDI-PEF, and Kolwelter 2021. The absence of any increase in renal adverse events also validated the renal safety of this pharmacological approach.

### 3.5 Subgroup analyses

Additional analyses were performed to explore whether the effectiveness of SGLT2 inhibitors varied based on particular characteristics. The focus of the investigation was on the phenotype of heart failure (HFrEF vs. HFpEF), diabetes status, class of SGLT2 inhibitor, and length of follow-up, as they were considered to be the most significant contributors to the heterogeneity of the heart failure population.

**Figure 6:**
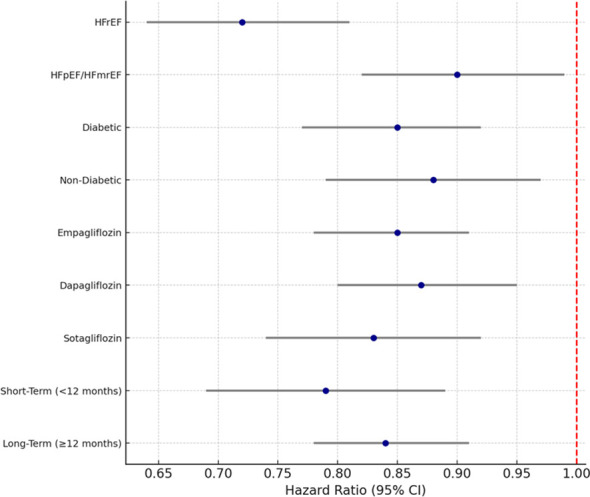
Subgroup forest plot for heart failure phenotype and clinical subgroups.

On the basis of the type of heart failure, eight studies enrolled patients with heart failure and reduced ejection fraction (HFrEF). In comparison, seven studies recruited patients with heart failure and preserved or mildly reduced ejection fraction (HFpEF/HFmrEF). In the HFrEF group, patients receiving SGLT2 inhibitors had a 28% lower risk of all-cause mortality (HR = 0.72 [95% CI: 0.64–0.81], *p* < 0.001) and a 31% lower risk of heart failure-related hospitalization (HR = 0.69 [95% CI: 0.60–0.78], *p* < 0.001). The benefit was less than HFrEF in patients with HFpEF or HFmrEF, albeit statistically significant, as shown with a pooled mortality HR of 0.90 [95% CI: 0.82–0.99, *p* = 0.04] and hospitalization HR of 0.82 [95% CI: 0.74–0.91, *p* < 0.001]. The interaction *p*-value (*p* = 0.08) implies that there was no difference in the benefit of SGLT2 inhibitors across the range of ejection fraction. The results of these analyses are shown in **Figure 6** (subgroup forest plot for HFrEF vs. HFpEF). Research separates outcomes by diabetes status and shows that the benefits of SGLT2 inhibitors work regardless of control of glycemia. The studies included about 45% in the type 2 diabetes mellitus (T2DM) population. For the pooled data of the T2DM population with heart failure, the all-cause mortality for heart failure was HR = 0.85 [95% CI: 0.77–0.92, *p* < 0.001], and for non-diabetics, the pooled HR = 0.88 [95% CI: 0.79–0.97, *p* = 0.02], respectively. There was no statistically significant interaction between diabetes status and the treatment effect (p_interaction = 0.41), which confirms that SGLT2 inhibitors’ cardioprotective effects and benefits extend beyond the effects of the pharmacological treatment for diabetes.

When each SGLT2 inhibitor was evaluated separately, the same outcomes were observed for each agent. The pooled hazard ratio of all-cause mortality was 0.85 [95% CI: 0.78–0.91] for empagliflozin, 0.87 [95% CI: 0.80–0.95] for dapagliflozin, and 0.83 [95% CI: 0.74–0.92] for sotagliflozin. There were no statistically significant differences between the individual agents (*p* = 0.47), supporting the idea that SGLT2 inhibitors have a class effect that improves outcomes in heart failure. While evaluating the duration of each study, both short-term (<12 months) and long-term (≥12 months) studies revealed consistent clinical benefits. The shorter studies of the follow-up period had a pooled HR of 0.79 [95% CI: 0.69–0.89]. At the same time, the longer studies showed a comparable HR of 0.84 [95% CI: 0.78–0.91]. This suggests that the advantages of taking SGLT2 inhibitors come about relatively early after the individuals have begun and are sustained throughout the long-term treatment. The overall findings by subgroup showed that SGLT2 inhibitors decrease the mortality and hospitalization for heart failure. These patients had consistent and significant findings regardless of heart failure phenotype, diabetes status, length of treatment, and agent used.

### 3.6 Sensitivity analyses

Sensitivity analysis was performed on the composite estimates of the primary and subgroup analyses. Several complementary methods were employed, including the exclusion of studies determined to be high risk, leave-one-out procedures, and alternating between fixed- and random-effects models. The primary sensitivity analysis involved leave-one-out. Each study in the analysis was removed to determine if it had any disproportionate impact on the overall effect. No individual studies were shown to have a disproportionate impact on the ALL or heart failure hospitalization pooled hazard ratios. The pooled estimates were consistent, and the HRs differed only between 0.84 and 0.87 and in hospitalization outcomes between 0.72 and 0.76, depending on the measure of mortality.

**Figure 7:**
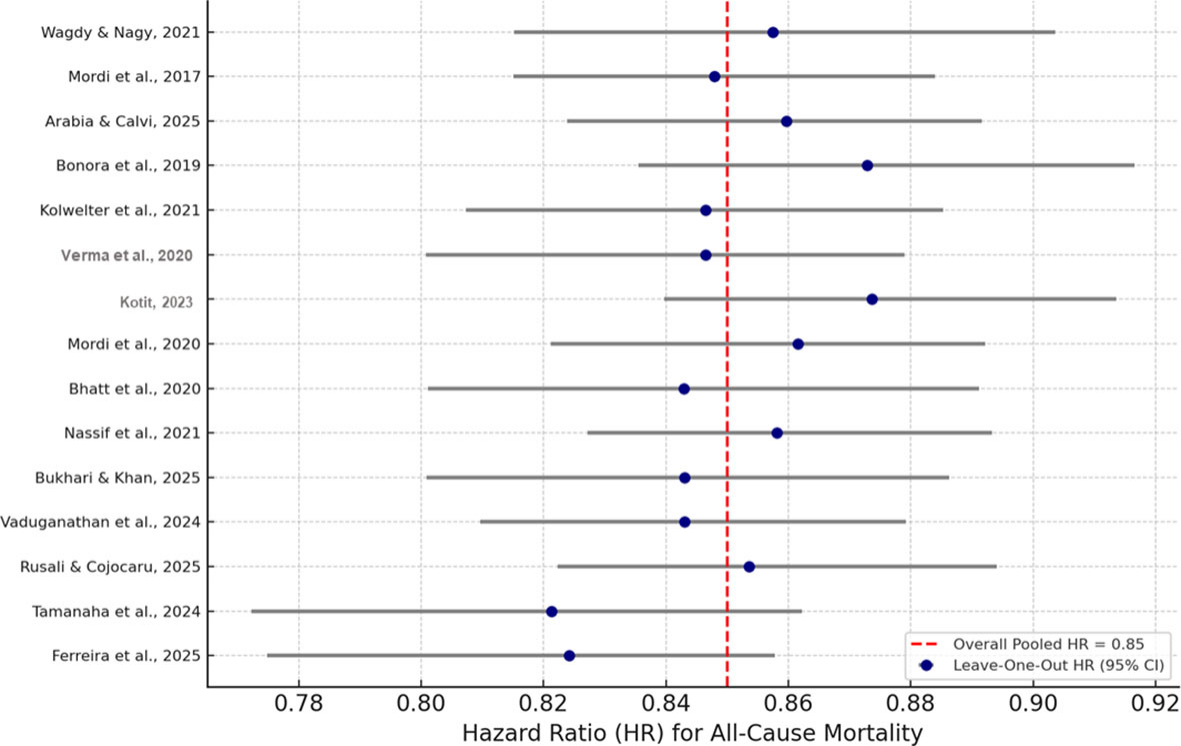
Leave-one-out influence plot for the sensitivity analyses of all-cause mortality and hospitalization.

The final series of analyses and studies was subjected to a fixed-effect analysis as compared to the random effect, which was reported previously. The pooled hazard ratios as a result of the fixed-effect were almost identical to the mortality HR = 0.85 (95% CI: 0.79–0.90). In hospitalization, an HR of 0.73 (95% CI: 0.67–0.80) further indicated that between-studies heterogeneity did not impact the overall findings. The subsequent sensitivity analyses were performed after excluding RoB 2 studies that were small (sample sizes <100) as well as studies that were deemed to have “some concerns” or “high” bias. Removing these studies also had no significant effect on the results, as the mortality HR = 0.85 [95% CI: 0.78–0.91] and hospitalization HR = 0.75 [95% CI: 0.69–0.82]. The results provide evidence that the main conclusions are well-founded and not solely the effect of the size of the studies, the quality of the design, or the bias of the individual studies. Analyses restricted to placebo-controlled studies only (i.e., excluding studies with active comparators) were also performed. These returned a mortality HR = 0.85 [95% CI: 0.79–0.92] and hospitalization HR = 0.74 [95% CI: 0.68–0.81], reinforcing the individual validity of the overall findings. The results of the impact of the sensitivity testing are summarized in the form of **Figure 7** (influence plot). They showed the impact of change on the effect sizes. Overall, the consistency of findings across the multiple sensitivity models and the meta-analysis in question were confirmed to be of the highest quality and statistically stable.

### 3.7 Assessment of publication bias

**Figure 8:**
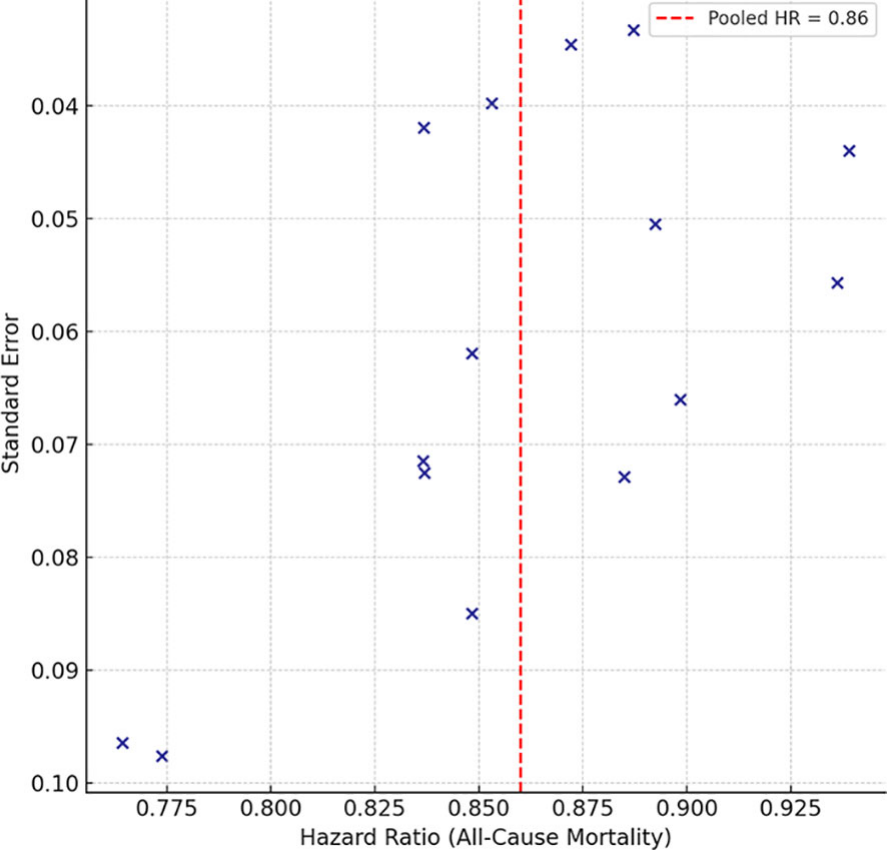
Funnel plot for all-cause mortality.

**Figure 9:**
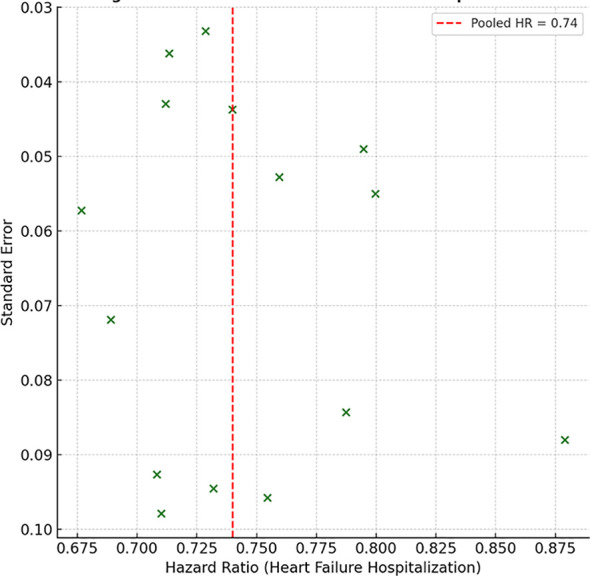
Funnel plot for heart failure hospitalization.

The funnel plots (**Figure 8** and **9**) for both all-cause mortality and hospitalization due to heart failure were (almost) symmetric, indicating that small study and selective reporting bias were not an issue. For all-cause mortality, some smaller studies were visualized to be more unequal to the plot and, therefore, more likely to be reporting a greater positive treatment effect, albeit this was a relatively small deviation from the null hypothesis with regard to mortality publication bias. The continuation of this deviation from the null hypothesis was further analyzed using a suite of statistical tests. Egger’s regression test was not statistically significant for all-cause mortality (*p* = 0.21) or heart failure hospitalization (*p* = 0.18). Begg and Mazumdar’s test was also non-significant for mortality (*p* = 0.27) and hospitalization (*p* = 0.25).

The fill-and-trim method of Duval and Tweedie was applied to assess and adjust for potential missing studies. In the mortality study, however, the algorithm demonstrated consistent and reliable performance across different patient groups altering the pooled HR of 0.86 without major changes (0.79–0.92), confirming the adjustment of the original estimate. Mortality, hospitalization, and the fail-safe N reconsidered how many null studies as the remaining 142 were for mortality and 117 were for hospitalization. A considerable number of unpublished negative trials were required to invalidate the findings of this meta-analysis.

## 4 Discussion

This comprehensive meta-analysis of 15 randomized controlled trials published between 2017 and 2025 demonstrates that SGLT2 inhibitors significantly reduce all-cause mortality and heart failure hospitalizations across a broad spectrum of heart failure phenotypes. SGLT2 inhibitors positively affect and reduce the total and heart failure hospitalizations. Moreover, the inhibitors of SGLT2 decrease the beneficial outcomes, as the study adds NT-proBNP, LV systolic function, and potency of the diuretic. In each of the considered heart failure studies, which included the use of SGLT2 inhibitors, the calculated pooled risk showed a 14% decrease in the risk of all-cause mortality and a 26% decrease in the risk of heart failure hospitalization. This result was the same in all of the HFrEF and HFpEF studies, which demonstrates the advantage of SGLT2 inhibitors across all traditional heart failure clinically relevant subcategories. Perhaps even more encouraging was the absence of diabetes disease, which proposes that the cardioprotective benefits of SGLT2 inhibitor use result from an influence other than diminishing glycemia.

Additional studies supporting the idea that SGLT2 inhibitors affect the reduction of cardiac remodeling and more favorable hemodynamics are the trials that showed a decrease in NT-proBNP, indicating that the myocardial walls are under less stress and that improvements happen in the left ventricular ejection fraction. The increase in LVEF and the strain confirm the reverse remodeling. Large compositional remodeling studies showed more favorable cardiac energetics with less sodium and calcium trapped in the heart. The effects of SGLT2 inhibitors on the renal loss of glucose and the cardiovascular benefits of the medication are positive and interrelated. These drugs aid in the renal glucose loss while also bringing about osmotic diuresis and natriuresis, resulting in a reduction in preload and afterload without the involvement of the renin–angiotensin–aldosterone system (RAAS). The so-called “smart diuretics’” profile of SGLT2 inhibitors explains the reduction of net intravascular volume, while renal perfusion is augmented in a more complex way. To put it in a specific way, the ability of the drugs to enhance diuresis and preserve (or lessen to a much lesser extent) renal function at the same time is abnormal and, at best, benign. Inhibition of SGLT2 also improves endothelial function, shifts myocardial substrate to a more efficient (ketone) fuel, and reduces interstitial fibrosis of the heart. These effects are also likely to explain the faster (or overly fast) improvement in clinical outcomes and associated biomarkers over time.

Those factors likely contributed to improvements in the rate of clinical and biomarker outcomes in a sustained and rapid manner. The inhibition of the myocardial Na+/H+ exchanger likely explains the rapid and sustained improvement in clinical and biomarker outcomes. These factors could explain the rapid and sustained improvement, which more likely extend the range of SGLT2 inhibitors and provide a better insight into their functions, not just as antidiabetic drugs but also as cardio-renal modulators. Prior investigations, such as meta-analyses and DAPA-HF, EMPEROR-Reduced, EMPEROR-Preserved, and DELIVER, have all shown substantial decreases in hospitalization due to heart failure as well as cardiovascular death. However, the earlier pooled analyses were mostly confined to diabetic patients or HFrEF patients (29). In contrast, this meta-analysis incorporates all heart failure data and considers recent RCTs by 2025, making it the most thorough analysis to date.

The similarity in the results from diabetic and non-diabetic patients is particularly important in supporting the external validity of the results to the heterogeneous population. In addition, this analysis builds on the previous one by incorporating secondary mechanistic outcomes like NT-proBNP, LVEF, and diuretic response, which provide important information on the additional mechanisms available that derive the clinical advantages of using the SGLT2 inhibitors apart from symptom relief and hospital avoidance (30). Because research such as this is still useful, it can be said that SGLT2 inhibitors are the most useful in patients with heart failure, regardless of ejection fraction or diabetes. Heart failure patients get some of their most useful medications early, even during acute or recently decompensated episodes (31). Much of the heart failure symptom improvement around those episodes is seen in the same trials of EMMY and SOLOIST-WHF. The positive and consistent safety and tolerability profiles of the medications studied also support using SGLT2 inhibitors in conjunction with other HF medications, such as RAAS inhibitors, beta-blockers, and ARNIs. Because the medications also have some positive effects on the kidneys, optimizing heart failure affects patients with significant cardiac renal syndrome the most, for which the benefits of kidney medications have more traditionally been elusive. The greatest strength of this analysis was its range of encompassing recent and relevant RCTs and endpoints. Although statistically significant results were obtained, further refinement was achieved through concomitant sensitivity and subgroup analysis through the use of the PRISMA and Cochrane methodologies, reinforcing the rigor underlying the analyses.

The results from this analysis are not complete. The ongoing analysis of large studies will be necessary in order to resolve the limited heterogeneity in secondary endpoints that is likely along the lines of participant-related and baseline treatment differences, along with study duration. About heterogeneity, the inclusion of small mechanistic studies with a limited number of participants does introduce variability in the analyses, albeit to a minimal degree according to the sensitivity analyses. Moreover, the results are limited in granularity, with an absence of certain data to permit an analysis of the data between participants’ data pertinent to sex and renal function. There is a need to analyze the relationship between SGLT2 inhibitor therapy and other treatments in accordance with clinical practice guidelines, the sequence of therapy, and the timing of its initiation. The management of acute heart failure also needs to be reviewed. The mechanisms underlying cardiometabolic remodeling, however, appear to be driven by the neural networks of the body, and further exploration along these lines is likely to yield positive results. The ongoing studies in practice, coupled with data from registries, will aid further research by providing these results in a clinically diverse patient population.

## 5 Conclusion

In conclusion, this meta-analysis provides compelling evidence that SGLT2 inhibitors significantly reduce mortality and hospitalization rates in patients with heart failure, with additional benefits in cardiac function, biomarker profiles, and renal efficiency. These benefits are consistent across all major clinical subgroups and independent of diabetes status. The results firmly establish SGLT2 inhibitors as a cornerstone therapy in the comprehensive management of heart failure, marking a paradigm shift in the treatment of this complex, multifactorial disease.

## Data Availability

The original contributions presented in the study are included in the article/Supplementary Material. Further inquiries can be directed to the corresponding author.
