# Supplementary material for: Glucose cotransporter-2 inhibitors on mortality and hospitalization in heart failure patients: a comprehensive meta-analysis
Source: Front Endocrinol (Lausanne). 2026 May 13;17:1758519. doi: 10.3389/fendo.2026.1758519 (PMC13212064; doi:10.3389/fendo.2026.1758519)
Supplement: Supplementary file 1 [file Table1.docx]

**Supplementary Tables**

**Supplementary Table S1:**

**Trial-Level Mapping of Included Studies to Outcomes, Effect Measures, Follow-up Duration, and Subgroup Availability**

| **Trial (Year)** | **HF Phenotype** | **HF Etiology** | **Endpoints Contributed to Meta-analysis** | **Effect Measure Used** | **Follow-up Duration** | **Subgroup Data Available** |
| --- | --- | --- | --- | --- | --- | --- |
| Wagdy & Nagy, 2021 (EMPEROR-Preserved) | HFpEF | Mixed | All-cause mortality; HF hospitalization; NT-proBNP | HR (mortality, hospitalization); MD (NT-proBNP) | 26 months | Diabetes status; EF range |
| Mordi et al., 2017 (RECEDE-CHF) | HFrEF | Mixed | NT-proBNP; Diuretic efficiency | MD (NT-proBNP); MD (urine output) | 6 months | Diabetes (100% T2DM) |
| Arabia & Calvi, 2025 | HFrEF | Mixed | LVEF; Reverse remodeling | MD (LVEF %) | 12 months | Not reported |
| Bonora et al., 2019 | HFpEF | Mixed | LV systolic function; NT-proBNP | MD (LVEF); MD (NT-proBNP) | 8 months | Diabetes (100% T2DM) |
| Kolwelter et al., 2023 | HFrEF | Mixed | NT-proBNP; Diuretic response | MD (NT-proBNP); MD (natriuresis) | 6 months | Not reported |
| Ganguly, 2024 (DELIVER-like) | HFpEF/HFmrEF | Mixed | All-cause mortality; HF hospitalization; NT-proBNP | HR (mortality, hospitalization); MD (NT-proBNP) | 28 months | Diabetes; EF subgroup |
| Dougherty et al., 2023 | HFrEF (Post-MI HF) | Ischemic | All-cause mortality; HF hospitalization; LVEF | HR (mortality, hospitalization); MD (LVEF) | 12 months | Post-MI subgroup |
| Mordi et al., 2020 | HFrEF | Mixed | Diuretic efficiency; NT-proBNP | MD (urine output); MD (NT-proBNP) | 3 months | Diabetes (100% T2DM) |
| Bhatt et al., 2020 (SOLOIST-WHF) | HFrEF | Mixed | All-cause mortality; HF hospitalization | HR (mortality, hospitalization) | 12 months | Diabetes (100% T2DM) |
| Nassif et al., 2021 | HFpEF | Mixed | NT-proBNP; HF hospitalization | HR (hospitalization); MD (NT-proBNP) | 12 months | Diabetes status |
| Bukhari & Khan, 2025 | HFrEF | Mixed | All-cause mortality; HF hospitalization | HR (mortality, hospitalization) | 18 months | Diabetes status |
| Vaduganathan et al., 2024 | HFpEF | Mixed | All-cause mortality; HF hospitalization | HR (mortality, hospitalization) | 24 months | Background SGLT2i stratified |
| Rusali & Cojocaru, 2025 | HFrEF (Post-MI HF) | Ischemic | All-cause mortality; HF hospitalization | HR (mortality, hospitalization) | 9 months | Post-MI subgroup |
| Tamanaha et al., 2024 (LUCENT-J) | HFpEF | Mixed | LV function (MFR surrogate); NT-proBNP | MD (functional parameters) | 12 months | Diabetes status |
| Ferreira et al., 2025 | HFpEF | Mixed | LV systolic function; NT-proBNP | MD (LVEF); MD (NT-proBNP) | 12 months | Not reported |
